# Evaluating the Academic Influence of Orthopedic Surgeons in Spinal Literature Through Relative Citation Ratio

**DOI:** 10.7759/cureus.25147

**Published:** 2022-05-19

**Authors:** Zachary T Grace, Harsh Patel, Ali M Omari, Angeline Sanders, Nareena Imam, John D Koerner

**Affiliations:** 1 Orthopaedics, Hackensack Meridian School of Medicine, Nutley, USA; 2 Orthopaedics, Rothman Orthopaedics, Paramus, USA; 3 Office of Clinical Research Administration, Hackensack University Medical Center, Hackensack, USA

**Keywords:** sciatica, radiculopathy, neurogenic claudication, lumbar spinal stenosis, lumbar disc herniation, cervical disc herniation, spine, orthopedics, relative citation ratio

## Abstract

Introduction: The innovative iCite tool applies the relative citation ratio (RCR) to gauge the time and field-adjusted scientific influence of a publication. This study examines scholarly effects on spine surgery to distinguish the impact made by orthopedic surgeons, neurosurgeons, and several other specialists.

Materials and methods: From 2013 to 2017, 100 of the highest RCR-rated articles were gathered for each of the following terms: cervical disc herniation (CDH), lumbar disc herniation (LDH), lumbar spinal stenosis (LSS), neurogenic claudication (NC), radiculopathy (RAD), and sciatica (SC). The first, second, and last authors were queried for background and academic qualifications and placed into the following specialty categories: orthopedic surgery, neurosurgery, pain management, medicine, and others. To provide an alternative degree of influence, the Scopus database was employed to classify the h-index associated with each author.

Results: Across the six search terms, there were 526 orthopedic surgeons among 1,730 authors (30.4%), with the highest representation in LSS (118/290, 40.7%), and the lowest in SC (45/286, 15.7%). Orthopedics was the most influential specialty across all six research terms by median RCR (p = 0.012). Compared to their neurosurgical counterparts, orthopedic authors had a greater influence in CDH (3.93 vs. 2.63, p = 0.0492), LDH (5.10 vs. 4.99, p = 1.0000), NC (2.16 vs. 1.40, p = 0.2370), and SC (3.35 vs. 3.04, p = 0.5285), but had a lower influence in LSS (5.13 vs. 5.32, p = 0.7736) and RAD (5.03 vs. 6.05, p = 0.3938).

Conclusion: Orthopedic surgeons lead other specialties when determining scholarly influence through RCR across six of the pre-designated research domains within spine surgery. For orthopedics, a modest influence in LSS and RAD may suggest potential areas of future focus. The use of bibliometrics to analyze available literature enables us to identify other specialties that have contributed to our field and promote interdisciplinary collaboration.

## Introduction

Academic influence in medicine can be studied through published literature to examine how medical professionals, such as orthopedic surgeons, contribute to their respective fields. Classically, a publication’s impact has been quantified using the journal impact factor (JIF). JIF is calculated over a two-year period by dividing the number of times articles published in a journal were cited by the total number of articles published in that journal [1]. Unfortunately, the broad use of JIF undermines the effect individual studies have in a specific field of interest.

The impact of literature has previously been analyzed by ranking the most influential articles that exist to acknowledge their contribution to evidence-guided clinical decision-making. For spine surgery, in particular, rankings have been created for distinct topics, including the top 100 cervical spinal surgery articles and the top 100 lumbar spondylolisthesis articles [2,3]. These rankings typically rely on a citation index’s reporting of the “times cited” per article, such as the Thomson Reuters Web of Science.

The use of bibliometrics (objective statistical analyses to measure scholarly output) such as the JIF has now become universally recognized as the way to compare publications, authors, and institutions on their academic impact, rather than looking at the raw number of times cited [4]. However, these bibliometric indicators do have some flaws despite their usefulness (such as inflation from self-citation). As a result, flawed metrics can be detrimental to the reputation of a journal and its editors. This can lead to the loss of quality of manuscript submissions to a journal, creating a subsequent further negative impact on a journal's future citation impact [5].

The development of the relative citation ratio (RCR) by the National Institute of Health (NIH) has been shown to be a reliable metric to analyze an article’s weight compared to its respective peer publications [6]. Among other bibliometrics, the RCR is reported for NIH-funded publications in the NIH’s iCite database [6]. The iCite database provides a monthly-updated view of different bibliometrics for such publications. The RCR is a ratio of its article citation rate (ACR) to its normalized field citation rate (FCR) [7]. The ACR represents the number of times a paper was cited divided by the number of years since its publication [7]. The FCR represents the average of the journal citation rates for articles published in the same field [7]. Despite concerns over slight variability based on how fields are defined, the RCR is still regarded as an attractive and intuitive metric that has great potential use in academic medicine [7].

Additionally, analysis of the impact made by a subspecialty in a field would benefit from using an author-level metric as well. The h-index metric can provide information on the productivity and impact an author from a specific medical professional group has compared to their peers from another group [8]. By using an additional established metric for RCR such as the h-index, further comparisons across fields can be made through statistical analysis [6,8].

This study seeks to examine the scholarly impact of orthopedic surgeons and other specialists in spine surgery literature by using the aforementioned bibliometrics. We hypothesized that orthopedic surgeons would have a substantial impact on multiple domains of spinal literature; however, there will be some areas showing a more modest impact. Furthermore, we hypothesized that our colleagues will also have a considerable contribution to spinal literature.

## Materials and methods

To illustrate scholarly developments emerging in spine research, field-specific terms of the study were collated at the discretion of the principal investigator. These topics include a broad array of commonly recognized pathologies of the spine that were thought to encompass a majority of influential literature in the field. The identified topics included: “cervical disc herniation” (CDH), “lumbar disc herniation” (LDH), “lumbar spinal stenosis” (LSS), “neurogenic claudication” (NC), “radiculopathy” (RAD), and “sciatica” (SC). From 2013 to 2017, each research topic was analyzed using the NIH iCite web application to produce the highest RCR-rated results from the top 100 articles. This period of time was chosen specifically to allow for an up-to-date glimpse into the landscape of current trends in spinal literature, while still allowing enough time to pass for articles to gain sufficient citations.

The professional backgrounds of the first, second, and last authors of each study were analyzed using data gathered from an online query. The respective position of each author was weighted equally during the analysis. The authors were then classified into one of five specialty groups based on each individual’s respective training: orthopedic surgery, neurosurgery, pain management (including anesthesiologists and physical medicine and rehabilitation specialists), medicine (including family medicine, internal medicine, radiology, neurology, etc.), and other (non-physicians, doctor of philosophy, doctor of chiropractic, etc.).

The RCR was employed to gauge the time and field-adjusted scientific influence of a publication relevant to orthopedic spine surgery. The novelty of RCR allows for field-specific normalization for comparisons of different disciplines [9]. This approach is distinct from traditional bibliometric values in that RCR is based on the co-citation network of the article [1].

Additional research metrics, such as the h-index, were incorporated to allow for a more robust analytical discussion. Using the Scopus citation database from Elsevier, h-index values were gathered for each of the identified authors and subdivided among specialty groups. The h-index is a well-established, individual-specific metric that attempts to capture the citation impact and productivity of the author [10]. It is defined as a value of *h* such that the author has published *h* articles cited at least *h* times [11].

Statistical methods

All data from the iCite and Scopus databases were compiled using Microsoft Excel (Microsoft Corporation, Redmond, WA). Normality of RCR and h-index were assessed using the Shapiro-Wilk test. Descriptive statistics were reported for each specialty overall and within each research term using medians for continuous variables and n (%) for categorical variables. To test differences between specialty groups, the Kruskal-Wallis test was performed followed by Dunn’s test with Holm’s adjustment. All analyses were performed in R version 3.6.2 (R Foundation for Statistical Computing, Vienna, Austria) with a p-value less than 0.05 indicating statistical significance.

## Results

Six research terms identified 1,730 authors, of which 525 (30.4%) were orthopedic surgeons. This specialty group comprised the highest percentage of authors in LSS (118/290, 40.7%), LDH (100/295, 33.9%), and RAD (97/291, 33.3%). Neurosurgeons were found to be the most represented specialty in the CDH (103/288, 35.8%) and NC (101/280, 36.1%). The final research term, sciatica, was most represented by the “other” specialty group (115/286, 40.2%). There was a marked decrease in the number of orthopedic (45/286, 15.7%) and neurosurgery (42/286, 14.7%) specialists represented in the sciatica domain (Table 1).

**Table 1:** Influential authorship and median RCR by author specialty ^a^ P-value reported from the Kruskal-Wallis test. * Indicates a p-value < 0.05. RCR, relative citation ratio; CDH, cervical disc herniation; LDH, lumbar disc herniation; LSS, lumbar spinal stenosis; NC, neurogenic claudication; RAD, radiculopathy; SC, sciatica.

Topic	Orthopedic authors	Orthopedic surgery	Neurosurgery	Medicine	Pain	Other	P-value^a^
Overall	30.4	4.52	4.17	4.35	4.15	4.28	0.0120*
CDH	26.0	3.93	2.63	3.14	3.03	2.88	0.0930
LDH	33.9	5.10	4.99	6.99	6.07	5.29	0.0686
LSS	40.7	5.13	5.32	4.76	6.47	5.20	0.0934
NC	32.1	2.16	1.40	1.36	1.01	1.11	0.0005*
RAD	33.3	5.03	6.05	5.49	4.60	6.46	0.0193*
SC	15.7	3.35	3.04	3.58	2.62	3.01	0.0467*

**Figure 1:**
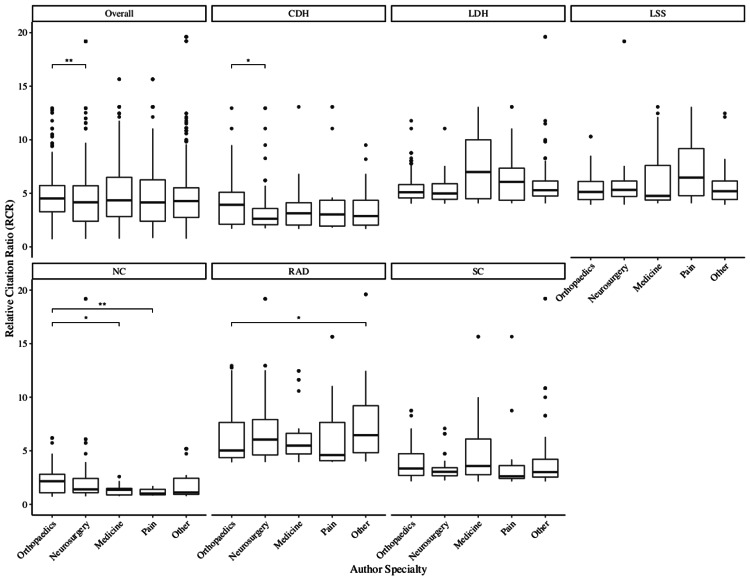
RCR values by author specialty Pairwise comparisons were conducted using Dunn’s test with Holm’s adjustment. * p < 0.05, ** p < 0.01, *** p < 0.001. Note that 11 data points were excluded from the figure for readability. RCR, relative citation ratio; CDH, cervical disc herniation; LDH, lumbar disc herniation; LSS, lumbar spinal stenosis; NC, neurogenic claudication; RAD, radiculopathy; SC, sciatica.

Across all six research terms, there were differences in RCR among the specialties (p = 0.0120). Orthopedic surgeons had the highest median RCR value, which was significantly higher than that of neurosurgeons (4.52 vs. 4.17, p = 0.0068) (Figure 1). Compared to their neurosurgical counterparts, orthopedic authors had a greater influence in CDH (3.93 vs. 2.63, p = 0.0492), LDH (5.10 vs. 4.99, p = 1.0000), NC (2.16 vs. 1.40, p = 0.2370), and SC (3.35 vs. 3.04, p = 0.5285), but had a lower influence in LSS (5.13 vs. 5.32, p = 0.7736) and RAD (5.03 vs. 6.05, p = 0.3938). Within the NC research term, orthopedic surgeons had a greater influence than those specializing in medicine (2.16 vs. 1.36, p = 0.0205) and pain (2.16 vs. 1.01, p = 0.0040). However, within the RAD topic, authors from the “other” specialty category dominated and had a greater impact than orthopedic surgeons (6.46 vs. 5.03, p = 0.0474).

The specialties and identified research terms were subjected to analysis through another bibliometric tool known as the h-index. Overall, there were differences in the h-index by specialty (p < 0.0001) (Table 2).

**Table 2:** Median h-index by author specialty ^a^ P-value reported from the Kruskal-Wallis test. * Indicates a p-value < 0.05. CDH, cervical disc herniation; LDH, lumbar disc herniation; LSS, lumbar spinal stenosis; NC, neurogenic claudication; RAD, radiculopathy; SC, sciatica.

Topic	Orthopedic surgery	Neurosurgery	Medicine	Pain	Other	P-value^a^
Overall	17.00	19.00	25.00	20.00	14.00	<0.0001*
CDH	16.00	16.00	22.50	28.00	13.50	0.0102*
LDH	14.00	19.00	45.00	22.00	21.00	0.0032*
LSS	18.00	23.00	48.50	38.00	19.00	0.0007*
NC	14.50	18.00	17.00	9.00	13.00	0.5383
RAD	19.00	20.00	19.50	20.00	12.50	0.3258
SC	16.00	17.50	24.50	8.50	14.00	0.0154*

**Figure 2:**
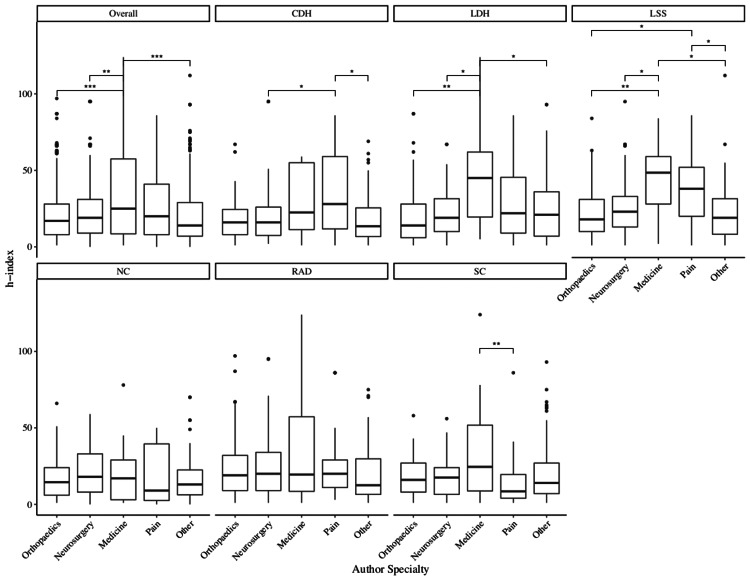
H-index values by author specialty Pairwise comparisons were conducted using Dunn’s test with Holm’s adjustment. * p < 0.05, ** p < 0.01, *** p < 0.001. Note that one data point was excluded from the figure for readability. CDH, cervical disc herniation; LDH, lumbar disc herniation; LSS, lumbar spinal stenosis; NC, neurogenic claudication; RAD, radiculopathy; SC, sciatica.

Across the six research terms, orthopedic authors held the fourth-highest median h-index out of the five specialty groups, significantly lower than medicine, the highest-rated group (17.0 vs. 25.0, p < 0.0001). Those specializing in medicine also had a greater impact than neurosurgeons (25.0 vs. 19.0, p = 0.0042) and “other” specialty authors (25.0 vs. 14.0, p < 0.0001). However, there were differences between the two bibliometrics. With respect to h-index, orthopedic surgeons had the same median in CDH compared to neurosurgery (16.0 vs. 16.0, p = 0.9816), but smaller influence in the other five domains, although none of the comparisons were statistically significant: LDH (14.0 vs. 19.0, p = 0.5943), LSS (18.0 vs. 23.0, p = 1.0000), NC (14.5 vs. 18.0, p = 1.0000), RAD (19.0 vs. 20.0, p = 0.7718), and SC (16.0 vs. 17.5, p = 1.0000) (Figure 2). In both the LDH and LSS terms, orthopedic surgeons had less impact than those in medicine (14.0 vs. 45.0, p = 0.0014 and 18.0 vs. 48.5, p = 0.0076), respectively.

## Discussion

Traditional bibliometric indices such as the h-index reveal orthopedics did not achieve the highest median specialty value in any of the six research terms. However, RCR analysis indicated orthopedics attained the highest significant median RCR for the overall and NC categories among the five specialty groups. Potential opportunities to focus orthopedic research efforts through RCR include the RAD and SC research domains.

These results could be explained by patient preference in regards to seeing either an orthopedic surgeon or a neurosurgeon for a specific pathology. If patients are more frequently consulting with an orthopedic surgeon for radiculopathy, for example, there will be a higher likelihood of research being performed on this pathology. Patient preference for selecting an orthopedic surgeon versus a neurosurgeon for specific pathologies may also influence the ability to perform research due to inadequate sample sizes. Patients may also initially see their primary care physician for these pathologies, which often resolve with conservative treatment, and therefore surgical consultation is not needed.

The lesser contributions of orthopedic surgeons to the RAD and SC research domains may be due to the effectiveness of non-surgical management options for these conditions. For cervical radiculopathy, non-surgical treatments such as anti-inflammatory medications and physical therapy have been proven effective as evidenced by 75-90% of patients achieving symptomatic improvement with non-operative measures [12,13]. Sciatica management draws several similarities to radiculopathy in the implementation of physical therapy, anti-inflammatory medications, and corticosteroid injections in some instances [14]. Typically, these therapies show significant resolution of symptoms within four to six weeks with no long-term complications [15]. While the studied research terms with a strong orthopedic presence may also have non-operative treatment measures, RAD and SC could be subject to increased contributions from other specialty groups adding valuable insight that can inform the delivery of care.

In showcasing differing strengths of orthopedic influence through two separate bibliometrics, it is important to note the limitations of possible self-citation and the inability to account for the individual impact of different journals or different types of studies performed [16]. Specifically, it was noted during the development of the h-index that it can be subjective to compare multiple papers from different research topics to each other since the index does not account for the popularity of the topic [17].

Determination of academic success is often predicated on research productivity and grant funding [18]. The use of an author-specific (h-index) metric, as well as a publication-specific (RCR) metric, stratifies the level of analysis of the search term field among the related specialties [8]. Improvements in scholarly production, academic influence, research funding, and industry payments to healthcare providers can increase a surgeon’s and institution’s reputation, which, in turn, can influence patients’ decision-making in where to seek care [18]. Departmental improvement can also impact the financial compensation of orthopedic surgeons [19].

Orthopedic surgeons, especially spine surgeons, should be cognizant of journals and scholarly output by their colleagues particularly within neurosurgery and medicine. These highly influential articles may be overlooked or unnoticed if their audience is not appropriate based on the journal [20]. Therefore, orthopedic-specific journals should consider the possible benefit of publishing research performed by non-orthopedic surgeons. The opposite may also be true; orthopedists publishing in non-orthopedic journals may reach greater viewership and produce more influential articles.

Limitations of an objective bibliometric, such as RCR, may have influenced the final results of this study. The retrospective nature of data collection from the iCite database means that we are unable to obtain the most up-to-date information. Since the information was gathered over a set period, it is possible some values may have changed throughout the collection [21]. However, this is unlikely to cause significant bias, given the time period queried and the frequent monthly updating of the iCite database. Additionally, bias may result from the RCR only considering the number of times a publication has been cited while failing to account for the variation of journals in which it has been cited.

Aside from the usage of the aforementioned bibliometrics, the design of the study itself has limitations. The six fields of study (CDH, LDH, LSS, NC, RAD, and SC) were chosen with the scholarly judgment of the most relevant areas of focus, rather than with an established metric or guideline. Furthermore, retrieval of the authors from each publication included only three authors (first, second, and last) listed on the authorship string, and thus may have omitted important authors from other healthcare fields. However, the decision to use only these influential authors is in adherence to multiple institutional guidelines used for academic promotion via journal publications [22]. Most importantly, we acknowledge that academic medicine cannot solely be considered without clinical context when measuring the influence of specific fields on spinal literature. Varying outside factors including cost, time, skillset, and training are all to be recognized when analyzing intricate relationships between medical specialty and the target subject.

Statistical limitations

Due to small sample sizes within some comparisons, we had low power to detect differences of very small effect sizes. While we did observe differences in magnitude, they were not always statistically significant. To combat this, we reported all results and used the Holm method to adjust for multiple comparisons rather than more conservative methods like the Bonferroni method [23].

## Conclusions

Orthopedic surgeons lead other specialties when determining scholarly influence through an article-specific metric, RCR, across six of the pre-designated research domains within spine surgery, with opportunities to improve growth into RAD and SC domains. However, evaluation with the author-specific h-index metric reveals orthopedics ranked fourth out of the five specialties studied. These results illuminate the potential benefits of being cognizant of our colleagues’ research in spinal literature, such as finding areas for self-improvement and discovering new topics capable of interdisciplinary collaboration.

## References

[1] Hutchins BI, Yuan X, Anderson JM, Santangelo GM (2016). Relative citation ratio (RCR): a new metric that uses citation rates to measure influence at the article level. PLoS Biol.

[2] Skovrlj B, Steinberger J, Guzman JZ, Overley SC, Qureshi SA, Caridi JM, Cho SK (2016). The 100 most influential articles in cervical spine surgery. Global Spine J.

[3] Aldawsari K, Alotaibi MT, Alsaleh K (2020). Top 100 cited articles on lumbar spondylolisthesis: a bibliographic analysis. Global Spine J.

[4] Franchignoni F, Özçakar L, Negrini S (2018). Basic bibliometrics for dummies and others: an overview of some journal-level indicators in physical and rehabilitation medicine. Eur J Phys Rehabil Med.

[5] Huggett S (2013). Journal bibliometrics indicators and citation ethics: a discussion of current issues. Atherosclerosis.

[6] Ündar A (2017). The relative citation ratio: measuring impact of publications from an international conference with a new NIH metric. Artif Organs.

[7] Janssens AC, Goodman M, Powell KR, Gwinn M (2017). A critical evaluation of the algorithm behind the relative citation ratio (RCR). PLoS Biol.

[8] Rock CB, Prabhu AV, Fuller CD, Thomas CR Jr, Holliday EB (2018). Evaluation of the relative citation ratio, a new National Institutes of Health-supported bibliometric measure of research productivity, among academic radiation oncologists. J Am Coll Radiol.

[9] Surkis A, Spore S (2018). The relative citation ratio: what is it and why should medical librarians care?. J Med Libr Assoc.

[10] Nowak JK, Lubarski K, Kowalik LM, Walkowiak J (2018). H-index in medicine is driven by original research. Croat Med J.

[11] Bertoli-Barsotti L, Lando T (2017). The h-index as an almost-exact function of some basic statistics. Scientometrics.

[12] Woods BI, Hilibrand AS (2015). Cervical radiculopathy: epidemiology, etiology, diagnosis, and treatment. J Spinal Disord Tech.

[13] Childress MA, Becker BA (2016). Nonoperative management of cervical radiculopathy. Am Fam Physician.

[14] Koes BW, van Tulder MW, Peul WC (2007). Diagnosis and treatment of sciatica. BMJ.

[15] Davis D, Maini K, Vasudevan A (2022). Sciatica. StatPearls. Treasure Island.

[16] Bhandari M, Busse J, Devereaux PJ (2007). Factors associated with citation rates in the orthopedic literature. Can J Surg.

[17] Hirsch JE (2005). An index to quantify an individual's scientific research output. Proc Natl Acad Sci U S A.

[18] Stavrakis AI, Patel AD, Burke ZD, Loftin AH, Dworsky EM, Silva M, Bernthal NM (2015). The role of chairman and research director in influencing scholarly productivity and research funding in academic orthopaedic surgery. J Orthop Res.

[19] Buerba RA, Sheppard WL, Herndon KE (2018). Academic influence and its relationship to industry payments in orthopaedic surgery. J Bone Joint Surg Am.

[20] Starbuck WH (2005). How much better are the most-prestigious journals? The statistics of academic publication. Organ Sci.

[21] Davis FM, Obi AT, Gallagher KA, Henke PK (2020). Accessing the academic influence of vascular surgeons within the National Institutes of Health iCite database. J Vasc Surg.

[22] Mentzelopoulos SD, Zakynthinos SG (2017). Research integrity, academic promotion, and attribution of authorship and nonauthor contributions. JAMA.

[23] Holm S (1979). A simple sequentially rejective multiple test procedure. Scand J Stat.

